# Increasing RB1 Expression by Targeting EZH2 in Triple‐Negative Breast Cancer

**DOI:** 10.1111/jcmm.70384

**Published:** 2025-03-11

**Authors:** Renfei Yang, Liyan Fei, Yingfei Xue, Yu Zhang, Qian Hu, Lu Guo, Yong Wei, Qin Wu

**Affiliations:** ^1^ Hangzhou Institute of Medicine (HIM) Chinese Academy of Sciences Hangzhou China; ^2^ School of Chemistry and Material Science University of Science and Technology of China Hefei China; ^3^ School of Pharmaceutical Science and Technology (SPST) Tianjin University Tianjin China; ^4^ College of Pharmacy Zhejiang University of Technology Hangzhou China; ^5^ Hangzhou Institute for Advanced Study (UCAS) Chinese Academy of Sciences Hangzhou China

**Keywords:** enhancer, epigenetics, EZH2, RB1, TNBC

## Abstract

Loss of RB1 function represents a defining characteristic of triple‐negative breast cancer (TNBC) and is intricately associated with resistance to therapeutic interventions. In this study, we investigate the epigenetic mechanisms governing RB1 expression in TNBC. Employing a combination of bioinformatics analyses and experimental validations, we identified lysine histone methyltransferase EZH2 as a key upstream regulator of RB1 expression. EZH2 primarily mediates trimethylation of lysine 27 on histone H3 as the catalytic subunit of the Polycomb repressive complex 2 (PRC2) complex. Furthermore, our findings demonstrate that pharmacological inhibition of EZH2 leads to a significant upregulation of RB1 expression levels, mediated by enhanced enrichment of the activating histone marker H3K27ac at the RB1 enhancer region, as evidenced by ATAC‐sequencing and ChIP‐qPCR assays. These insights unveil a promising clinical avenue for combating RB1‐mediated drug resistance in TNBC through the strategic integration of epigenetic‐targeting agents.

## 1 Introduction

Breast cancer remains a prominent global health concern, being the most commonly diagnosed cancer and the second leading cause of female mortality according to the latest 2024 cancer statistics report [1]. Subtyping of breast cancer, based on oestrogen receptor (ER), progesterone receptor (PR) and human epidermal growth factor receptor 2 (HER2) expression levels, classifies it into Luminal‐A, Luminal‐B, HER2‐positive and triple‐negative breast cancer (TNBC) subtypes. TNBC, defined by the absence of ER, PR and HER2 expression, accounts for approximately 15%–20% of breast cancer cases and presents a significant clinical challenge due to its resistance to conventional endocrine and HER2‐targeted therapies [2]. TNBC patients typically face a distinct clinical profile characterised by earlier onset age, heightened metastatic potential, increased risk of recurrence and diminished overall survival rates compared to other breast cancer subtypes [3]. Recent studies have highlighted various molecular pathways involved in TNBC progression and resistance. For instance, ononin has been shown to inhibit TNBC lung metastasis by targeting the EGFR‐mediated PI3K/Akt/mTOR pathway [4]. Similarly, the methylation of GPRC5A promotes liver metastasis and docetaxel resistance by activating the mTOR signalling pathway in TNBC [5]. Moreover, the FOXO1‐induced LYPLAL1‐DT impedes TNBC progression by mediating the hnRNPK/β‐catenin complex [6]. These findings underscore the complexity and heterogeneity of TNBC, necessitating the exploration of novel therapeutic targets and strategies.

RB1, the first human tumour suppressor gene identified, plays a pivotal role in cell cycle regulation by interacting with the transcription factor E2F1, leading to G1‐S checkpoint arrest and suppression of tumorigenesis [7, 8]. RB1 dysfunction, resulting from mutations, deletions, transcriptional silencing or hyperphosphorylation, significantly contributes to cancer development, particularly in TNBC, where approximately 30% of patients exhibit loss or downregulation of RB1 expression [9, 10]. While genetic alterations have traditionally been considered the primary drivers of RB1 dysregulation, recent studies underscore the crucial role of epigenetic mechanisms [11, 12]. For instance, DNMT3A and SUV39H1 promote the methylation of CpG islands in the RB1 promoter, leading to reduced RB1 expression and enhanced cancer cell growth, as observed in melanoma [13]. Similarly, HDAC‐2 directly binds to the RB1 promoter in myeloid‐derived suppressor cells, silencing RB1 and impacting cell differentiation and immune responses [14]. EZH2, the catalytic subunit of PRC2, silences genes through histone H3 lysine 27 trimethylation (H3K27me3), affecting over 200 tumour suppressor genes [15, 16]. EZH2 inhibitors, such as Tazemetostat, have gained FDA approval for treating certain malignancies [17]. Notably, EZH2 has been implicated in regulating RB1, particularly in prostate cancer, where reduced expression of RB1 and TP53 leads to upregulation of EZH2, promoting lineage plasticity and resistance to enzalutamide [18, 19]. While these findings suggest a relationship between EZH2 and RB1 across various cancers, direct evidence of EZH2's role in regulating RB1 expression remains limited.

In this study, we uncover a significant inverse correlation between EZH2 and RB1 expression in TNBC. Treatment with the EZH2 inhibitor (Tazemetostat) robustly amplifies RB1 expression levels in TNBC cell lines. Subsequent ATAC‐seq data interrogation identifies an accessible chromatin region within the 17th intron of the RB1 gene, potentially serving as an enhancer region. Finally, ChIP experiments corroborate that EZH2 inhibition enhances the H3K27ac occupancy at the RB1 enhancer sequence, thereby enhancing RB1 expression.

## 2 Results

### 2.1 Epigenetic Mechanisms Contribute to Diminished RB1 Expression in TNBC

**FIGURE 1:**
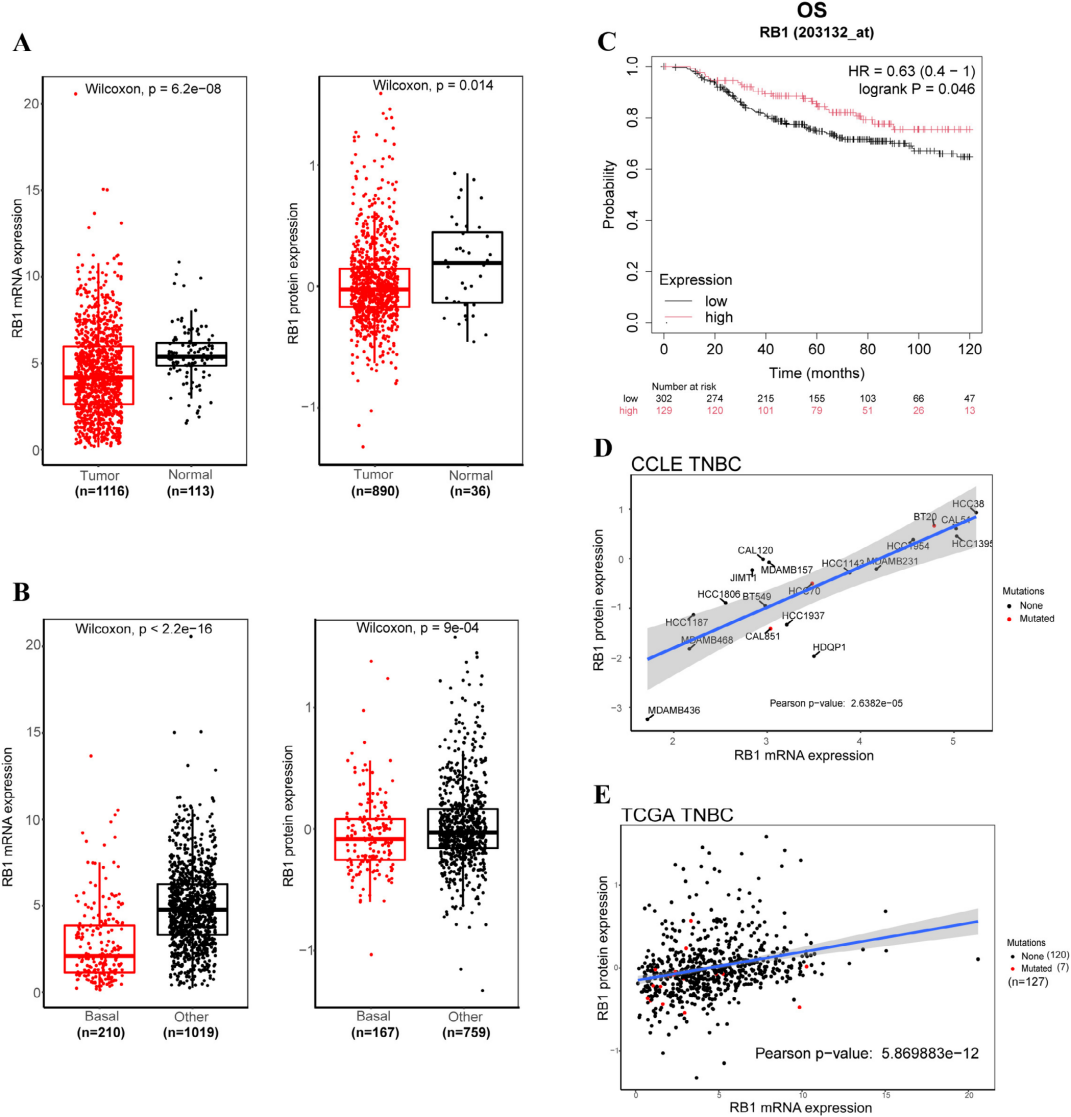
RB1 expression is low in TNBC. (A) RB1 expression levels in tumour tissues were compared to adjacent tissues using the TCGA datasets. (B) RB1 expression levels in the basal subtype were compared to other subtypes using the TCGA datasets. (C) Kaplan–Meier plot illustrates the overall survival (OS) of TNBC patients with high or low RB1 expression. (D, E) RB1 mRNA and protein expression levels were analysed in both CCLE (D) and TCGA (E) datasets. Black dots signify no mutation in the RB1 gene, while red dots indicate the presence of a mutation in the RB1 gene.

Evaluation of RB1 expression levels in TNBC revealed significant differences compared to adjacent non‐cancerous tissues. Both mRNA and protein levels of RB1 were notably higher in tumour samples than in non‐cancerous tissues (Figure 1A). However, within breast cancer subtypes, basal‐like breast cancer exhibited substantially lower RB1 mRNA and protein levels compared to other subtypes (Figure 1B). To ascertain the impact of RB1 expression levels on the prognosis of TNBC patients, we analysed the overall survival (OS) outcomes of basal‐like subtype (corresponding to the most common subtype of TNBC [20]) patients with different RB1 expression levels. Patients with low expression levels of RB1 demonstrate worse survival outcomes compared to those with high expression levels (Figure 1C). While prior research posited genetic or epigenetic factors as potential contributors to low RB1 expression, our analysis of the Cancer Cell Line Encyclopedia (CCLE) and The Cancer Genome Atlas (TCGA) databases unveiled a strong positive correlation between mRNA and protein levels of RB1 across both TNBC patient cohorts and cell lines, with mutations observed in only a minority of cases (Figure 1D,E). This compelling evidence suggests a pivotal role for epigenetic alterations in governing the reduced expression of RB1 in TNBC.

### 2.2 EZH2 Exhibits a Significant Negative Correlation With RB1 Expression

**FIGURE 2:**
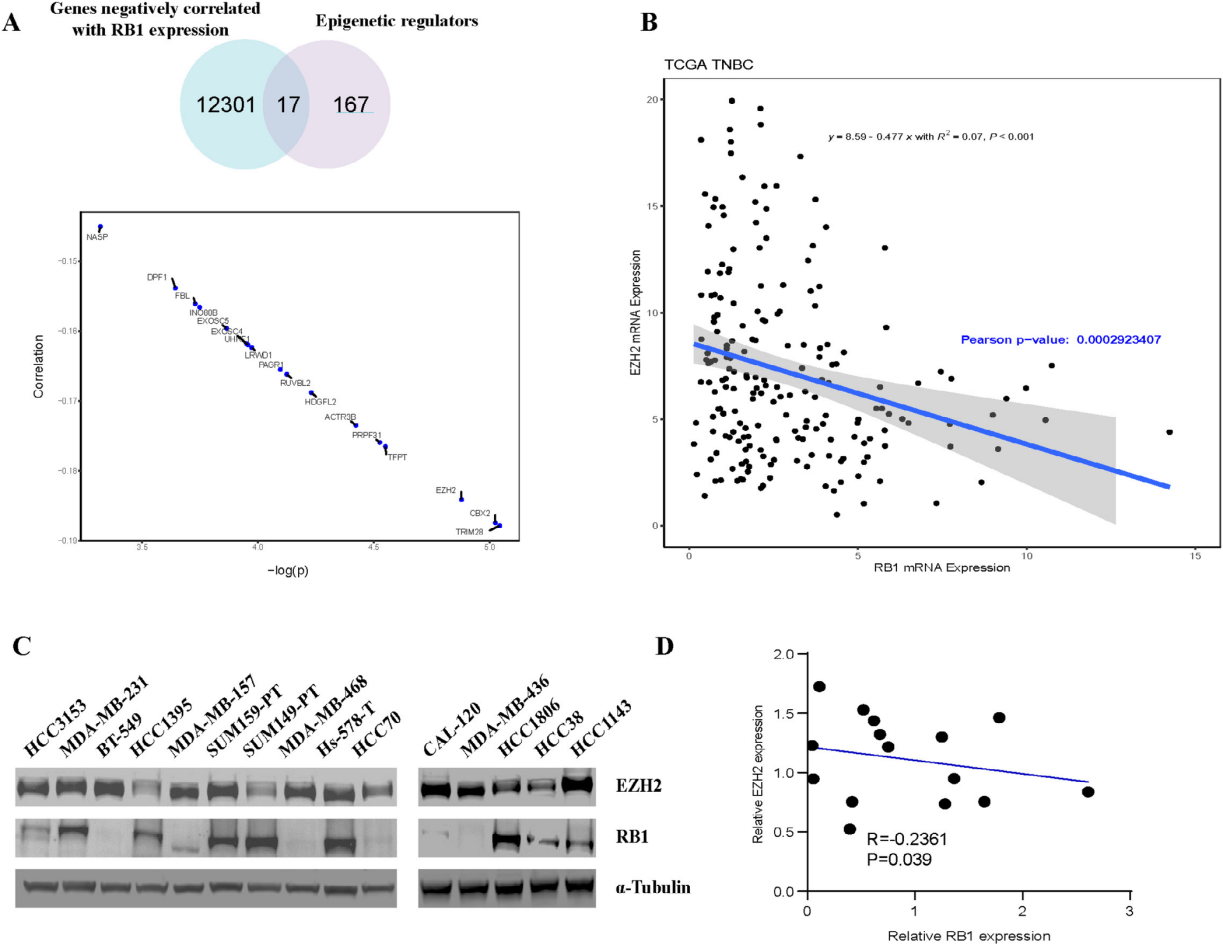
The negative correlation between EZH2 and RB1 in TNBC. (A) The Venn diagram illustrates 17 epigenetic regulatory proteins identified from the comparison of 12,301 genes negatively correlated with RB1 versus 167 epigenetic regulatory enzymes (above). The correlation diagram demonstrates that these 17 epigenetic regulatory enzymes exhibit a negative correlation with RB1 (below). (B) Transcriptional analysis from the TCGA database reveals a negative correlation between EZH2 and RB1. Each dot represents a TNBC patient. (C) Western blot analysis of EZH2 and RB1 expression in 15 TNBC cell lines. (D) Quantitative assessment of the results from (C) conducted using ImageJ.

To elucidate the epigenetic regulators implicated in the downregulation of RB1 in TNBC, we analysed all epigenetic regulatory enzymes negatively correlated with RB1 using the TCGA database, with EZH2 emerging as the third‐ranked candidate (Figure 2A). Foremost in our analysis ranks TRIM28, a complex protein comprising multiple subunits known for its role in facilitating histone deacetylase (HDAC) activity [21, 22]. Following TRIM28, CBX2, a subunit of Polycomb Repressive Complex 1 (PRC1), which recognises methylated histone H3, occupies the second position [23]. Within the CBX family, only CBX7 has small‐molecule inhibitors (MS37452, UNC3866) [24]. Conversely, EZH2, as the principal catalytic subunit of PRC2, stands out as a highly histone methyltransferase [25]. Numerous small molecule inhibitors targeting EZH2, including UNC1999, GSK126, GSK343, GSK926, EPZ005687, EPZ011989, CPI‐1205, CPI‐169 and others, have been developed, underscoring its potential for clinical translation [26]. Considering its potential in clinical translation, we selected EZH2 as the target of our subsequent research.

To investigate whether a negative correlation exists between EZH2 and RB1 expression, we examined their mRNA levels in TNBC patients using the TCGA database, unveiling a significant negative correlation (Figure 2B). Additionally, we assessed the protein levels of EZH2 and RB1 in 15 TNBC cell lines (Figure 2C), and quantitative analysis showed a negative correlation between their protein levels (Figure 2D). Collectively, our analysis of the TCGA patient database and our cell line data demonstrates a substantial inverse relationship between EZH2 and RB1 expression.

### 2.3 Suppression of EZH2 Augments RB1 Expression Levels

**FIGURE 3:**
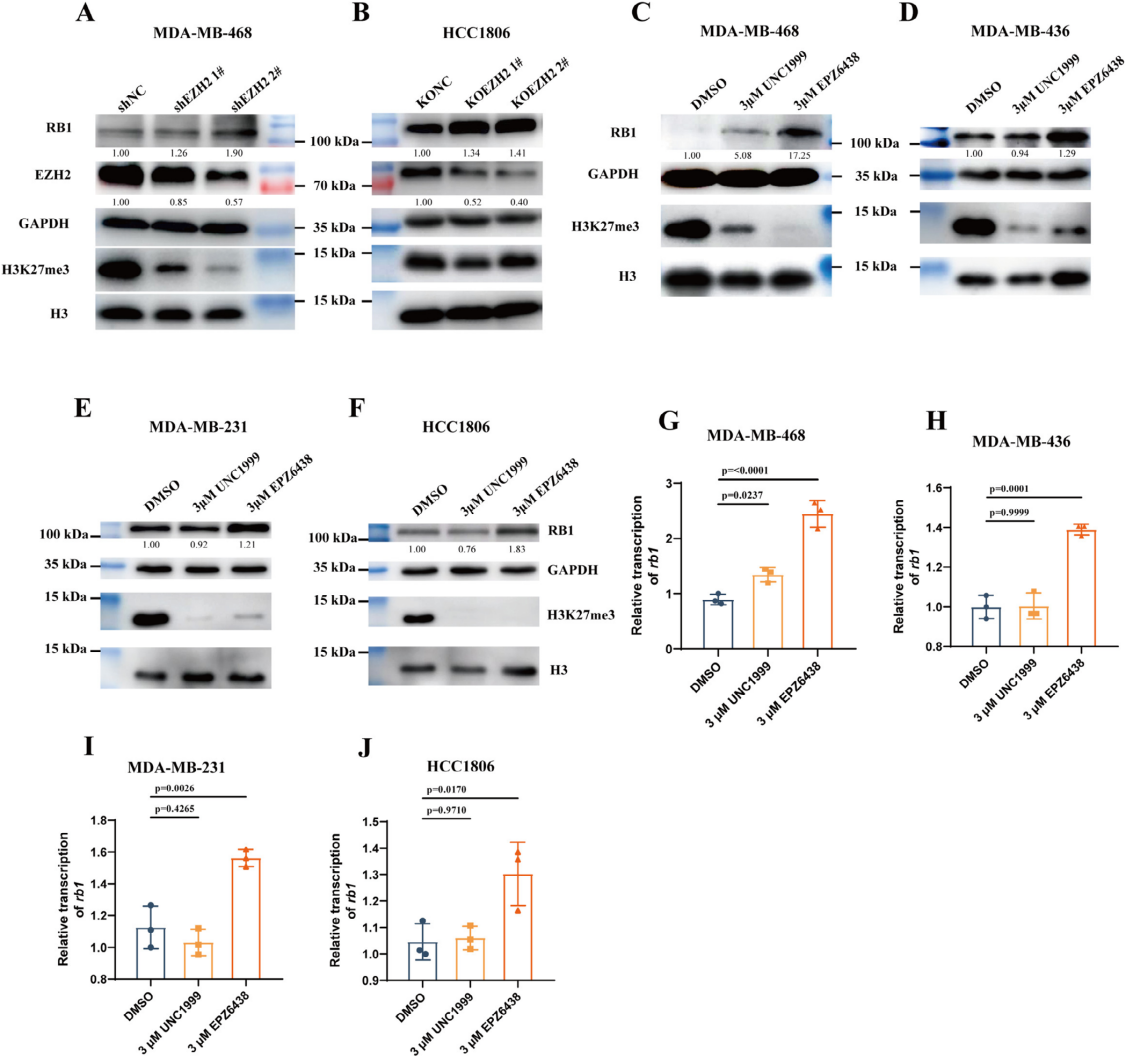
Inhibiting EZH2 improves RB1 expression. (A, B) Western blot analysis of EZH2, RB1 and H3K27me3 levels in MDA‐MB‐468 cells following shEZH2 treatment (A) and in HCC1806 cells after CRISPR‐KO of EZH2 (B). GAPDH and H3 were utilised as loading controls. (C–F) Western blot analysis of RB1 and H3K27me3 levels in MDA‐MB‐468 (C), MDA‐MB‐436 (D), MDA‐MB‐231 (E) and HCC1806 (F) cells post‐treatment with UNC1999 or EPZ6438. GAPDH and H3 served as loading controls. Quantitative analysis of the results was performed using ImageJ. (G–J) The relative mRNA levels of RB1 to GAPDH were determined in (G) MDA‐MB‐468, (H) MDA‐MB‐436, (I) MDA‐MB‐231 and (J) HCC1806 cells post‐treatment with UNC1999 or EPZ6438.

To validate the regulatory role of EZH2 on RB1, we utilised shRNA to knock down EZH2 in the RB1 low‐expressing cell line MDA‐MB‐468. Knocking down EZH2 in MDA‐MB‐468 led to a significant increase in RB1 expression levels (Figure 3A). Similarly, employing the CRISPR‐Cas9 system to knockout EZH2 in the high‐expressing RB1 cell line HCC1806 yielded analogous results (Figure 3B). These results indicate a pivotal role of EZH2 in the upstream regulation of RB1.

Considering the clinical relevance of EZH2, we explored the efficacy of several small molecule inhibitors targeting EZH2, including UNC1999, which targets both EZH1 and EZH2, and EPZ6438, specifically inhibiting EZH2. Two cell lines with low RB1 expression (MDA‐MB‐436 and MDA‐MB‐468) and two with high expression (MDA‐MB‐231 and HCC1806) were selected and treated separately with UNC1999 and EPZ6438. We observed a consistent elevation in RB1 expression levels following EPZ6438 treatment, but not UNC1999, irrespective of initial RB1 expression levels (Figure 3C–F). This suggests that inhibiting EZH1 has a certain counteracting effect on the regulatory function of EZH2 on RB1, indicating that the regulation system of RB1 expression involves multiple factors and is a complex process. Subsequent assessment of RB1 mRNA levels further substantiated that the observed increase in RB1 expression occurred at the transcriptional level (Figure 3G–J). These findings implicate EZH2 in the suppression of RB1 transcription through its methylation function at the H3K27 site, contingent upon the activity of the PRC2 complex.

### 2.4 Suppression of EZH2 Augments RB1 Expression by Enhancing H3K27ac Enrichment at the Enhancer Region

**FIGURE 4:**
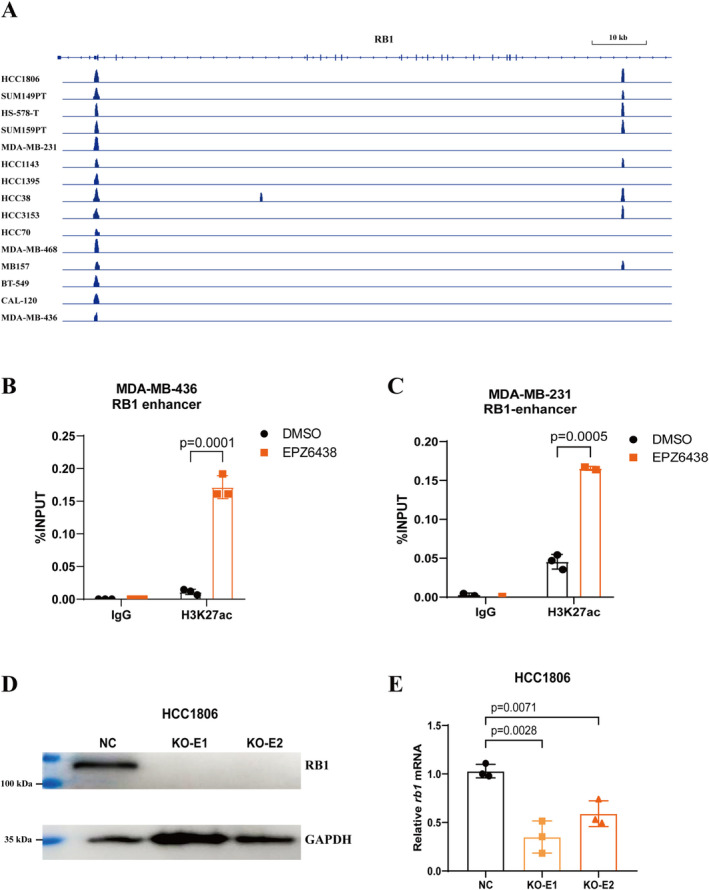
Inhibiting EZH2 elevates H3K27ac enrichment at the RB1 enhancer region. (A) ATAC‐seq tracks the RB1 gene locus in 15 TNBC cell lines, displaying chromatin openness using IgV visualisation software. The cell lines are arranged from top to bottom in descending order of RB1 expression levels, as shown in Figure 2C. (B, C) ChIP‐qPCR analysis of H3K27ac at the RB1 enhancer region in MDA‐MB‐436 (B) or MDA‐MB‐231 (C) after EPZ6438 treatment. (D) Western blot analysis of RB1 levels in HCC1806 cells with CRISPR knock‐out of the RB1 enhancer region. (E) Relative mRNA levels of RB1 to GAPDH in HCC1806 cells with CRISPR knock‐out of the RB1 enhancer region.

To further elucidate the mechanism underlying EZH2‐mediated regulation of RB1 expression, we initially investigated whether EZH2 directly methylates the promoter region of RB1 to suppress its expression. Analysing the occupancy of several major histone modifications in the promoter region of RB1 in MDA‐MB‐436 cells revealed a notable occupancy of H3K27ac, while H3K27me3 was conspicuously absent (Figure S1A). H3K4me3 is another target of EZH2, found at the RB1 promoter region. However, there are no significant differences in the occupancy levels of H3K4me3 among TNBC cell lines with varying levels of RB1 expression (Figure S1B). This suggests that EZH2 does not directly act on the promoter region of RB1 to regulate its expression. Subsequently, leveraging ATAC‐seq data from the previously analysed 15 TNBC cell lines, we categorised the openness status of the RB1 gene based on RB1 expression levels (Figure 4A). We identified an open region within the 17th intron of the RB1 gene, potentially serving as an enhancer in cell lines characterised by relatively high RB1 expression levels. To validate whether inhibiting EZH2 increases RB1 expression by modulating the accessibility of this enhancer region, we conducted ChIP‐qPCR analysis on MDA‐MB‐436 and MDA‐MB231 cells treated with EPZ6438. Strikingly, inhibition of EZH2 significantly increased the H3K27ac occupancy at this enhancer site in both RB1 low‐expressing and high‐expressing cell lines (Figure 4B,C). Furthermore, we utilised the CRISPR‐Cas9 system to knockout this enhancer sequence in the RB1 high‐expressing HCC1806 cell line, resulting in a notable reduction in both the protein and mRNA levels of RB1 (Figure 4D,E). In summary, our findings highlight an enhancer sequence within the 17th intron of the RB1 gene, through which EZH2 regulates the expression of RB1 by modulating the degree of enhancer accessibility. Inhibiting the methylation activity of EZH2 augments the enrichment of H3K27ac at this enhancer site, thereby enhancing the expression of RB1.

## 3 Discussion

Low RB1 expression not only contributes to tumour initiation and progression but also leads to resistance to various drug therapies. Apart from the previously mentioned resistance to androgen receptor antagonists in prostate cancer due to low RB1 expression [18], breast cancers with low RB1 levels also develop resistance to GLUT1 inhibitors [27]. Additionally, in the RB1–E2F pathway, CDK4/6 directly promotes RB1 phosphorylation, leading to its inactivation. Low RB1 expression is also a significant factor contributing to resistance against CDK4/6 inhibitors [28]. Our study has demonstrated that EZH2 inhibitors can elevate RB1 expression levels, suggesting that combining EZH2 inhibitors with other therapies could be a promising approach for treating cases characterised by low RB1 expression.

Inhibiting EZH2 activity leads to an increase in the occupancy level of H3K27ac at the enhancer site of RB1. This effect can be explained by the dynamic antagonistic relationship between H3K27me (including H3K27me1, H3K27me2 and H3K27me3) and H3K27ac. Loss of H3K27me3 due to EZH2 inhibition disrupts the transcriptional repression maintained by Polycomb group proteins, resulting in a corresponding increase in H3K27ac levels, which is associated with active gene transcription [29]. Studies have shown that this switch from methylation to acetylation of H3K27 is not only a hallmark of gene activation but also suggests a broader epigenetic reprogramming that enhances the transcriptional potential of target genes [30, 31]. This mechanism underscores the critical role of EZH2 in maintaining the balance between gene activation and repression through its modulation of histone marks, thereby offering insights into how EZH2 inhibitors might reactivate tumour suppressor genes like RB1. We hypothesised that inhibiting EZH2 would directly lead to a decrease in the H3K27me3 level at the RB1 enhancer, thereby increasing the H3K27ac level. However, our data did not indicate any alteration in the occupancy level of H3K27me3 at the RB1 enhancer upon EZH2 inhibition (data not shown). Therefore, inhibition of EZH2 methyltransferase activity indirectly increases the enrichment of H3K27ac at the RB1 enhancer.

TRIM28 stands out as the top epigenetic regulator inversely linked to RB1 expression levels. Functioning as a HDAC, TRIM28 plays a pivotal role in regulating tumour suppressor genes and enhancing transcription factor activity [32]. HDACs have emerged as a promising target in cancer chemotherapy research. Recent studies indicate that HDAC5 inhibitors boost RB1 expression and sensitise CDK4/6 inhibitors in prostate cancer cells [33]. Therefore, targeting TRIM28 could represent an alternative strategy to enhance RB1 expression.

In summary, we identified histone methyltransferase EZH2 as an upstream regulator of RB1 expression and confirmed that inhibiting EZH2 effectively increases RB1 expression. Through combined sequencing analysis, we elucidated that EZH2 regulates RB1 expression by enhancing the enrichment of the activating marker H3K27ac at the RB1 enhancer.

## 4 Methods and Materials

### 4.1 Cell Culture and Treatment

MDA‐MB‐231, MDA‐MB‐436 and MDA‐MB‐468 cell lines were grown in Dulbecco's modified Eagle's medium (Gibco, C11995500BT) supplemented with 10% fetal bovine serum (BIOIND, 04‐001‐01ACS) and 1% penicillin/streptomycin (Biosharp, BL505A). The HCC1806 cell line was grown in Roswell Park Memorial Institute 1640 (Gibco, C11875500BT) supplemented with 10% fetal bovine serum and 1% penicillin/streptomycin. All cells were maintained at 37°C with 5% CO_2_ (Thermo Fisher, 50145515) and were passaged no more than 25 times.

### 4.2 Small‐Interfering RNA (siRNA)‐Mediated Gene Knockdown

Cells were plated in six‐well plates at 2.0 × 10^5^ cells/well and cultured at 37°C in a 5% CO_2_ incubator for 24 h before transfection. Plasmid transfections were conducted using Hieff Trans (Yeasen, 40808ES03) according to the manufacturer's instructions. Target‐specific shRNA and non‐targeting control were purchased from Tsingke (Beijing, China) with the following target sequences: EZH2 shRNA 1#; 5′‐CCGGCGGCTCCTCTAACCATGTTTACTCGAGTAAACATGGTTAGAGGAGCCGTTTTTT‐3′, EZH2 shRNA 2#; 5′‐CCGGCCCAACATAGATGGACCAAATCTCGAGATTTGGTCCATCTATGTTGGGTTTTTT‐3′. CRISPR knock‐out plasmids P44551 (KO‐NC), P46722 (KO‐EZH2 1#) and P46818 (KO‐EZH2 2#) were purchased from MiaoLingPlasmid (www.miaolingbio.com). Cell samples were collected at a 48 h time point for qPCR or 96 h time point for WB.

### 4.3 CRISPR‐Cas9 System–Mediated Enhancer Region Knockout

Two gRNA sequences against the RB1 enhancer locus were designed with the use of the CRISPR Design method (http://crispr‐era.stanford.edu/show.jsp?g=0). The gRNA sequences were designed based on the RB1 enhancer region (chr13: 48974891–48975426, gRNA1: AAGACCCCGTTAAAATATGT; gRNA2: GAAGACCCCGTTAAAATATG). RB1 enhancer knock‐out vectors, containing either a non‐targeting control or RB1 enhancer gRNA/CRISPR/Cas9, were constructed using the pLentiCRISPRv2‐mCherry vector (Addgene #99154).

### 4.4 Western Blot

Cell samples were collected and lysed with lysis buffer (Beyotime, p0013) adding 1X protease inhibitor cocktail (Roche, 5056489001), 1X phosphatase inhibitor cocktail (Beyotime, P1045) and Benzonase (Beyotime, D7121‐25KU) on ice for 10 min. Proteins were quantified with BSA protein quantification kits (Beyotime, P0010). 20–40 μg of protein lysate extract were run on 10%–12% gradient gels and transferred to methanol‐activated PVDF membranes at 90 V for 2 h. Membranes were blocked with 5% skim milk for 1 h at RT. Primary antibodies used for membrane staining were anti‐RB1 (Abcam, ab181616; 1:1000), anti‐EZH2 (CST, 5246S; 1:1000), anti‐GAPDH (CST, 5174S; 1:1000), anti‐H3 (CST, 4499S; 1:1000), anti‐H3K27me3 (ABclonal, A19539; 1:1000). Membranes were stained with secondary antibodies (CST, 7076P2 or 7074P2) for 1 h at RT and imaged (AMERSHAM, IMAGE QUANT 800).

### 4.5 Real‐Time Quantitative PCR

Total RNA was extracted from cells or tissues using an RNA extraction kit (Vazyme, RC101). After cDNAs were transcribed (Yeasen, 11119ES60), gene expression was quantified by the SYBR Green Quantification Kit (Yeasen, 11184ES08) on the Lightcycler 96 (Roche). The amount of cDNA template was determined according to the instructions for the SYBR Green kit. The mixture was added to a white PCR reaction tube and placed in the Lightcycler 96. Data were collected on the B Lightcycler 96 and analysed by the LC96 Manager. All experiments were performed in at least three biological replicates. Primers used for this study were GAPDH (forward: TTCCAGGAGCGAGATCCCT, reverse: GGCTGTTGTCATACCTTCTCATGG) and RB1 (forward: GTGAACATCGAATCATGG, reverse: ATCAGTTGGTCCTTCTCG).

### 4.6 Chromatin Immunoprecipitation (ChIP)

For ChIP experiments, cells were seeded into 10‐cm plates at 30% confluence and treated with EZH2 inhibitor or siRNA. At 96 h after treatment, cells were washed once in 1X phosphate‐buffered saline (PBS) and then treated with 1% formaldehyde in PBS at 37°C for 10 min, followed by the addition of glycine to a final concentration of 0.125 M for 5 min. Cells were then scraped on ice and resuspended in 10 mL cell lysis buffer (5 mM PIPES (pH 8), 85 mM KCl, 0.5% NP40, 1X Complete Protease Inhibitor Cocktail proteinase inhibitors (Roche)) for 15 min. The nuclei were collected by centrifugation at 2000 g for 5 min at 4°C. The supernatant was carefully discarded and the pellet was resuspended in 1 mL of cold nuclear lysis buffer (50 mM Tris–HCl (pH 8), 10 mM EDTA, 0.8% sodium dodecyl sulphate (SDS), 1X Complete Protease Inhibitor Cocktail proteinase inhibitors (Roche)). The nuclei was sonicated with Covaris ME220 to make sure that most of the DNA is in pieces in the range of 200–500 bp. The IP reaction used 10 μg total chromatin and incubated it with 10 μg of anti‐H3K27ac or IgG antibody overnight at 4°C. Then 30 μL of the 50% protein G magnetic beads (CST, 9006S) was added and incubated for 2–4 h. The DNA was then purified using the Cycle Pure Kit (Omega, D6492) according to the manufacturer's instructions and analysed by RT‐qPCR. Prime sequences: RB1 enhancer: forward: AGTTCAGGGGGAAAAGGCATC, reverse: AGTGAACTAACACCACCCTCA.

### 4.7 Statistics and Reproducibility

TCGA and CCLE data analysis was conducted in R v.4.3.1. TCGA Gene expression and mutational data used for the analysis were accessed from USCS Xena under cohort GDC TCGA Breast Cancer (BRCA) under HTseq—counts and MuSE Variant Aggregation and Masking, respectively [34]. Expression data in counts format were then transformed into transcripts per million (TPM) using the *countToTpm_matrix* function from the R package GeoTcgaData v0.2.4. Expression comparisons and correlations were conducted using gene expression TPM units and protein expression normalised RPPA units. CCLE data were accessed from the DepMap portal (https://depmap.org/portal), and the DepMap Public 21Q4 dataset was selected for this analysis [35]. In gene expression comparisons, no inherit distributions were assumed for gene expression. Thus, a nonparametric Wilcoxon rank‐sum test was performed to determine statistical significance between groups using the package ggpubr v0.6.0. In expression correlation comparisons, the Pearson correlation coefficient and significance were determined using the default package stats v4.3.1. Survival Kaplan–Meier plots were constructed using the online tool Kaplan–Meier plotter (https://kmplot.com/) [36]. All 50 available datasets under the mRNA gene chip section were selected for comparison. Sample groups were split using the auto select option, a period of 120 months and basal PAM50 subtype were selected, while all other options were kept as default.

## Author Contributions

**Renfei Yang:** conceptualization (equal), data curation (equal), formal analysis (equal), funding acquisition (equal), investigation (equal), methodology (equal), project administration (equal), supervision (equal), writing – original draft (equal), writing – review and editing (equal). **Liyan Fei:** conceptualization (equal), formal analysis (equal), investigation (equal), methodology (equal), resources (equal), software (equal). **Yingfei Xue:** data curation (equal), investigation (equal). **Yu Zhang:** conceptualization (supporting), formal analysis (equal), methodology (equal). **Qian Hu:** data curation (equal), validation (equal). **Lu Guo:** data curation (equal), validation (equal). **Yong Wei:** conceptualization (equal), data curation (equal), methodology (equal), project administration (equal), resources (equal), writing – review and editing (equal). **Qin Wu:** formal analysis (equal), funding acquisition (equal), investigation (equal), methodology (equal), project administration (equal), resources (equal), supervision (equal), writing – review and editing (equal).

## Conflicts of Interest

The authors declare no conflicts of interest.

## Supporting information


**Figure S1.** EHZ2 do not regulate RB1 at the promoter region.

## Data Availability

The data that support the findings of this study are available on request from the corresponding author. The data are not publicly available due to privacy or ethical restrictions.
