# Supplementary material for: Increasing RB1 Expression by Targeting EZH2 in Triple‐Negative Breast Cancer
Source: J Cell Mol Med. 2025 Mar 11;29(5):e70384. doi: 10.1111/jcmm.70384 (PMC11897054; doi:10.1111/jcmm.70384)
Supplement: Supplementary file 1 — Figure S1. EHZ2 do not regulate RB1 at the promoter region. [file JCMM-29-e70384-s001.docx]

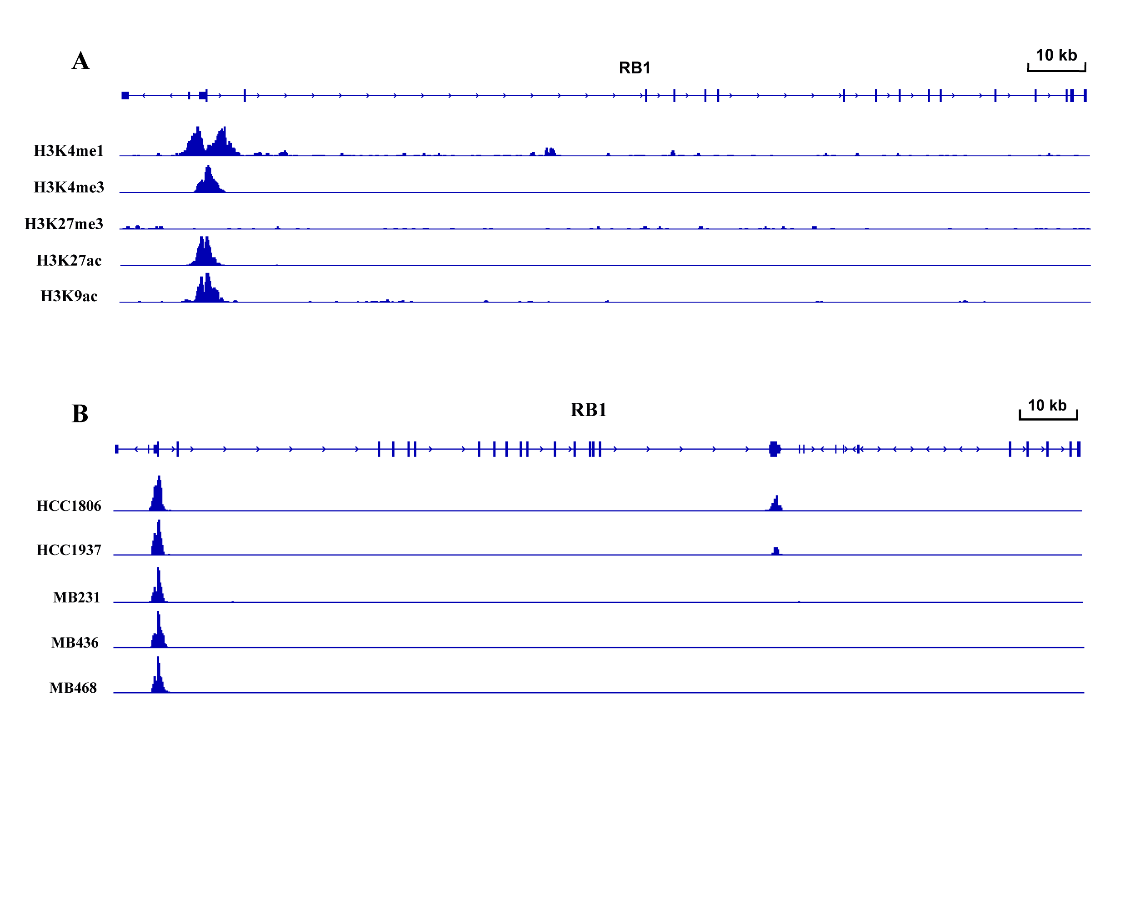


**Fig S1. EHZ2 do not regulate RB1 at promoter region.** (A) ChIP-seq assay of several forms of histone modifications in MDA-MB-436. (B) ChIP-seq assay of H3K4me3 in 5 TNBC cell lines with different expression levels of RB1.
